# Presentations of major peripheral arterial disease and risk of major outcomes in patients with type 2 diabetes: results from the ADVANCE-ON study

**DOI:** 10.1186/s12933-016-0446-x

**Published:** 2016-09-02

**Authors:** Kamel Mohammedi, Mark Woodward, Yoichiro Hirakawa, Sophia Zoungas, Stephen Colagiuri, Pavel Hamet, Stephen Harrap, Neil Poulter, David R. Matthews, Michel Marre, John Chalmers, on behalf of the ADVANCE Collaborative Group

**Affiliations:** 1 The George Institute for Global Health, University of Sydney, PO Box M201, Missenden road, Camperdown, Sydney, NSW 2050 Australia; 2 The George Institute for Global Health, University of Oxford, Oxford, UK; 3 Department of Epidemiology, Johns Hopkins University, Baltimore, MD USA; 4 Monash Centre for Health Research and Implementation, School of Public Health and Preventive Medicine, Monash University, Clayton, VIC Australia; 5 Boden Institute of Obesity, Nutrition, Exercise and Eating Disorders, Sydney Medical School, University of Sydney, Sydney, NSW Australia; 6 Research Centre, Centre Hospitalier de l’Université de Montréal, Montreal, Canada; 7 The University of Melbourne and Royal Melbourne Hospital, Melbourne, VIC Australia; 8 The International Centre for Circulatory Health, National Heart and Lung Institute, Imperial College, London, UK; 9 Oxford Centre for Diabetes, Endocrinology and Metabolism, National Institute for Health Research, Oxford Biomedical Research Centre, Harris Manchester College, University of Oxford, Oxford, UK; 10 INSERM, UMRS 1138, Centre de Recherche des Cordeliers, Paris, France; 11 Department of Diabetology, Endocrinology and Nutrition, Assistance Publique-Hôpitaux de Paris, Bichat Hospital, DHU FIRE, Paris, France; 12 Université Paris Diderot, Sorbonne Paris Cité, UFR de Médecine, Paris, France

**Keywords:** Type 2 diabetes, Peripheral arterial disease, Lower-extremity ulceration, Lower-extremity amputation, Mortality, Cardiovascular diseases, Major macrovascular events, Diabetic retinopathy

## Abstract

**Background:**

Peripheral arterial disease (PAD) is known to be associated with high cardiovascular risk, but the individual impact of PAD presentations on risk of macrovascular and microvascular events has not been reliably compared in patients with type 2 diabetes. We aimed to evaluate the impact of major PAD, and its different presentations, on the 10-year risk of death, major macrovascular events, and major clinical microvascular events in these patients.

**Methods:**

Participants in the action in diabetes and vascular disease: PreterAx and DiamicroN modified-release controlled evaluation (ADVANCE) trial and the ADVANCE-ON post-trial study were followed for a median of 5.0 (in-trial), 5.4 (post-trial), and 9.9 (overall) years. Major PAD at baseline was subdivided into lower-extremity chronic ulceration or amputation secondary to vascular disease and history of peripheral revascularization by angioplasty or surgery.

**Results:**

Among 11,140 participants, 516 (4.6 %) had major PAD at baseline: 300 (2.7 %) had lower-extremity ulceration or amputation alone, 190 (1.7 %) had peripheral revascularization alone, and 26 (0.2 %) had both presentations. All-cause mortality, major macrovascular events, and major clinical microvascular events occurred in 2265 (20.3 %), 2166 (19.4 %), and 807 (7.2 %) participants, respectively. Compared to those without PAD, patients with major PAD had increased rates of all-cause mortality (HR 1.35, 95 % CI 1.15–1.60, p = 0.0004), and major macrovascular events (1.47 [1.23–1.75], p < 0.0001), after multiple adjustments for region of origin, cardiovascular risk factors and treatments, peripheral neuropathy markers, and randomized treatments. We have also observed a trend toward an association of baseline PAD with risk of major clinical microvascular events [1.31 (0.96–1.78), p = 0.09]. These associations were comparable for patients with a lower-extremity ulceration or amputation and for those with a history of peripheral revascularization. Furthermore, the risk of retinal photocoagulation or blindness, but not renal events, increased in patients with lower-extremity ulceration or amputation [1.53 (1.01–2.30), p = 0.04].

**Conclusions:**

Lower-extremity ulceration or amputation, and peripheral revascularization both increased the risks of death and cardiovascular events, but only lower-extremity ulceration or amputation increased the risk of severe retinopathy in patients with type 2 diabetes. Screening for major PAD and its management remain crucial for cardiovascular prevention in patients with type 2 diabetes (ClinicalTrials.gov number, NCT00949286).

**Electronic supplementary material:**

The online version of this article (doi:10.1186/s12933-016-0446-x) contains supplementary material, which is available to authorized users.

## Background

Peripheral arterial disease (PAD) is a public health problem across the world with a significant impact on healthcare and a high economic burden [1–5]. It is associated with an increased risk of cardiovascular disease [6–8], and is particularly common in patients with type 2 diabetes [9–12]. Previous studies have shown poor survival and cardiovascular outcomes in patients with both type 2 diabetes and PAD [13–15]. However, the individual impact of different presentations of major PAD on survival and risk of major macrovascular and microvascular events in patients with type 2 diabetes has not been reliably evaluated in long-term prospective studies. The Action in Diabetes and Vascular Disease: PreterAx and DiamicroN Modified-Release Controlled Evaluation (ADVANCE) trial followed patients with type 2 diabetes for a median of 5.0 years, and the ADVANCE-ON post-trial observational study followed them up for further 5.4 years, to a median overall period of 9.9 years [16, 17]. The current investigation aimed to examine the impact of major PAD at baseline on the risk of mortality and major macrovascular and microvascular outcomes in patients with type 2 diabetes across the full 9.9 years, and to compare the effects on these outcomes, of presentation with lower-extremity ulceration or amputation on one hand, as against presentation with previous peripheral revascularization on the other.

## Methods

### Population study

ADVANCE was an international randomized trial in 11,140 patients with type 2 diabetes with the objectives to test the effect of intensive glucose control and blood pressure treatment on the incidence of major microvascular and macrovacular events [16]. Participants with type 2 diabetes at high risk of cardiovascular events were randomly assigned to a gliclazide (modified release)–based intensive glucose-control regimen, aiming to achieve an HbA1c ≤6.5 %, or to standard glucose control, with targets and regimens based on local guidelines. Participants were also randomly assigned to a fixed-dose combination of perindopril (4 mg) and indapamide (1.25 mg) or matching placebo. Subsequently 8494 surviving participants were enrolled in the post-trial observational evaluation, ADVANCE-ON, study. Design, characteristics of participants, and the main results of both studies have been previously published [16–19].

### Definition of peripheral arterial disease

Major PAD at baseline was defined, as in the ADVANCE study, as lower-extremity chronic ulceration (at least 6 weeks) or amputation below the knee (of at least one toe), secondary to vascular disease, or history of peripheral revascularization procedure by angioplasty or surgery [16]. Two presentations of major PAD were compared: lower-extremity ulceration or amputation versus peripheral revascularization. Patients with both presentations (n = 26) were excluded in the comparison of the outcomes according to each PAD presentation.

### Definition of outcomes

There were three primary outcomes: all-cause mortality, major macrovascular events (a composite of nonfatal myocardial infarction, nonfatal stroke, or cardiovascular death), and major clinical microvascular events (a composite of end-stage renal disease (ESRD), defined as requirement for renal-replacement therapy; death induced by renal disease; requirement for retinal photocoagulation; or diabetes-related blindness in either eye). The secondary outcomes were cardiovascular death, fatal or nonfatal myocardial infarction, fatal or nonfatal stroke, ESRD or renal death, and requirement for retinal photocoagulation or blindness. Outcomes were adjudicated by an independent End Point Adjudication Committee in the ADVANCE trial, through to the end of randomized treatment, and were reported by investigators without adjudication in the ADVANCE-ON study, in accord with its pre-specified protocol [17]. Information about the occurrence of study outcomes and of all serious adverse events was reported at the time of occurrence between visits. When study outcomes or serious adverse events occured, the responsible investigator of each centre ensured that the event was reported immediately by completing a serious adverse events form. The data and safety monitoring committee regularly reviewed all such events for each centre.

### Statistical analyses

Categorical variables were presented as the number of patients with the corresponding percentage. Continuous variables were expressed as mean (SD) or median (interquartile range) for those with skewed distribution. Characteristics of participants according to major PAD status (absence vs. presence) and presentation (lower-extremity ulceration or amputation vs. peripheral revascularization) were compared at baseline using Chi squared, ANOVA, or Wilcoxon tests. Cumulative incidence curves were used to plot survival (outcome-free) rates during follow-up. Survival curves were compared using the log-rank test. Cox proportional hazards survival regression models were fitted to examine the effect of baseline history of major PAD and its presentations on time-related survival (outcome-free) rates. Hazard ratios (HR), with associated 95 % confidence intervals (CI), were estimated according to major PAD status at baseline. Basic (model 1: region of origin, sex, age, body mass index, systolic blood pressure, history of ever smoking and study allocations), and full (model 2: model 1 plus duration of diabetes, HbA1c, waist circumference, heart rate, diastolic blood pressure, disturbance of 10-g monofilament sensation, absence of ankle and knee reflexes, estimated glomerular filtration rate (eGFR) [and its square for macrovascular analyses], total-, and HDL-cholesterol, triglycerides, use of antihypertensive, lipid lowering and antiplatelet drugs, and history of current alcohol drinking) adjustments were performed. Participants with a history of macrovascular disease (defined as the presence at baseline, of myocardial infarction, stroke, coronary artery bypass graft, percutaneous transluminal coronary angioplasty, hospital admission for unstable angina or transient ischaemic attack) were excluded in sensitivity analyses. p < 0.05 was considered as significant. Statistical analyses were performed using SAS software, version 9.3 (SAS Institute, http://www.sas.com).

## Results

### Clinical characteristics at baseline

Among 11,140 patients randomized at baseline, 516 (4.6 %) had a history of major PAD. Clinical characteristics of participants at baseline are shown in Additional file 1: Table S1. Participants with major PAD at baseline, compared to those without PAD, were older, and more frequently men and from established market economies. They had higher body mass index, waist circumference, and urinary albumin–creatinine ratio, and lower diastolic blood pressure, eGFR, and serum total and HDL-cholesterol. They were also more likely to use lipid lowering and antiplatelet drugs, and to have a history of ever smoking or current alcohol drinking. Patients with major PAD had also more frequent disturbance of 10-g monofilament sensation and absence of ankle and knee reflexes.

### Incidence of outcomes during follow-up according to the history of major PAD at baseline

**Fig. 1:**
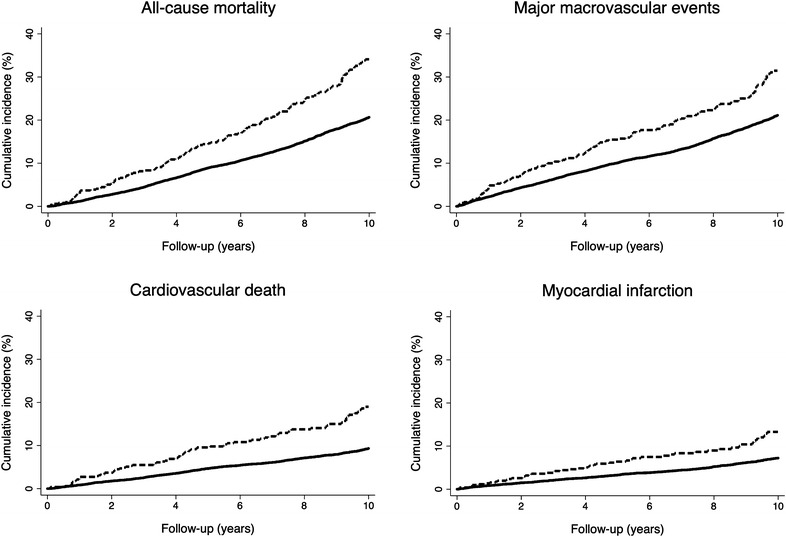
Cumulative incidence of all-cause mortality, major macrovascular events, cardiovascular death, and fatal or nonfatal myocardial infarction according to the absence (*solid line*) or the presence of major PAD (*dashed line*) at baseline (p < 0.0001 for all)

The median (interquartile interval) duration of overall follow-up was 9.9 (5.6–10.9) years. All-cause mortality, major macrovascular events, cardiovascular death, and major clinical microvascular events occurred in 2265 (20.3 %), 2166 (19.4 %), 988 (8.9 %), and 807 (7.2 %) participants, respectively. The cumulative incidence of all-cause mortality, major macrovascular events, cardiovascular death, and fatal or non-fatal myocardial infarction were higher in participants with history of major PAD at baseline compared to those without PAD (p < 0.0001 for all): Fig. 1; Table 1. Significant associations of PAD with the risk of all-cause mortality (HR 1.35, 95 % CI 1.15–1.60, p = 0.0004), major macrovascular events (HR 1.47, 95 % CI 1.23–1.75, p < 0.0001), cardiovascular death (HR 1.75, 95 % CI 1.39–2.21, p < 0.0001), and myocardial infarction (HR 1.58, 95 % CI 1.19–2.09, p = 0.001) persisted after adjustment for all potential confounding variables. A trend toward an association of major PAD with requirement of retinal photocoagulation or blindness persisted in the fully adjusted model (HR 1.39, 95 % CI 0.99–1.95, p = 0.05). However, we did not observe association of major PAD at baseline with the risk of ESRD or renal death (Table 1).

**Table 1:** Hazard ratios for outcomes during follow-up by history of major PAD at baseline

	Baseline history of major PAD		Hazard ratios (major PAD vs. not)					
	No (n = 10,624)	Yes (n = 516)	Model 1			Model 2		
			HR	95 % CI	p	HR	95 % CI	p
All-cause mortality, n (%)	2101 (19.8)	164 (31.8)	1.57	1.33–1.84	<0.0001	1.35	1.15–1.60	0.0004
Major macrovascular events, n (%)	2025 (19.1)	141 (27.3)	1.59	1.34–1.88	<0.0001	1.47	1.23–1.75	<0.0001
Cardiovascular death, n (%)	904 (8.5)	84 (16.3)	1.99	1.59–2.49	<0.0001	1.75	1.39–2.21	<0.0001
Myocardial infarction, n (%)	665 (6.3)	57 (11.0)	1.76	1.34–2.31	<0.0001	1.58	1.19–2.09	0.001
Stroke, n (%)	924 (8.7)	44 (8.5)	1.24	0.92–1.68	0.16	1.19	0.87–1.62	0.28
Major clinical microvascular events, n (%)	763 (7.2)	44 (8.5)	1.43	1.05–1.94	0.02	1.31	0.96–1.78	0.09
Retinal photocoagulation or blindness, n (%)	632 (5.9)	37 (7.2)	1.48	1.06–2.07	0.02	1.39	0.99–1.95	0.05
End-stage renal disease or renal death, n (%)	160 (1.5)	8 (1.6)	1.15	0.56–2.34	0.71	0.96	0.46–1.97	0.90

Model 1: adjusted for region of origin, sex, age, body mass index, systolic blood pressure, history of ever smoking, and study allocations. Model 2: adjusted as in model 1 plus duration of diabetes, HbA1c, waist circumference, heart rate, diastolic blood pressure, disturbance of 10-g monofilament sensation, absence of ankle and knee reflexes, estimated glomerular filtration rate (and its square for macrovascular analyses), total-, and HDL-cholesterol, triglycerides, use of antihypertensive, lipid lowering and antiplatelet drugs, and history of current alcohol drinking. p < 0.05 was significant

### Incidence of outcomes during follow-up according to different presentations of major PAD at baseline

**Fig. 2:**
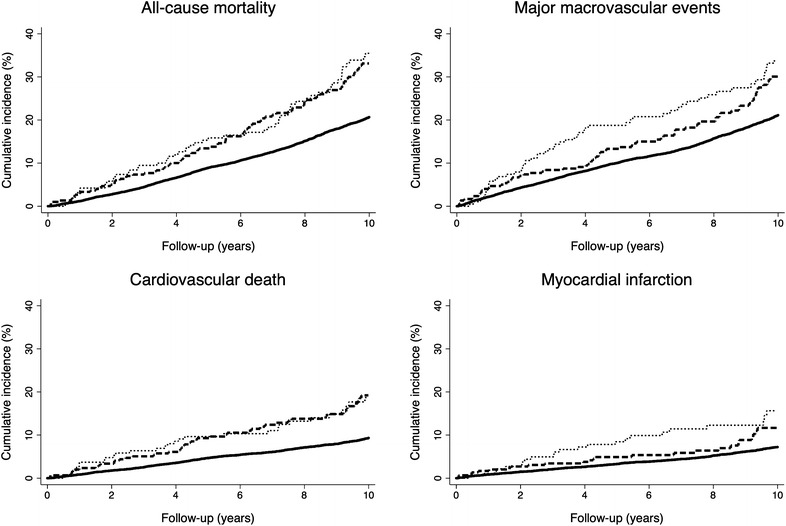
Cumulative incidence of all-cause mortality, major macrovascular events, cardiovascular death, and fatal or nonfatal myocardial infarction according to the absence (*solid line*) or the presence of lower-extremity ulceration or amputation (*dashed line*) and peripheral revascularization (*dotted line*) at baseline (p < 0.0001 for all)

Lower-extremity ulceration or amputation, and history of peripheral revascularization, not concurrently occurring, were established at baseline in 300 (2.7 %) and 190 (1.7 %) participants, respectively (p < 0.0001). Only 26 (0.2 %) patients had both presentations of major PAD at baseline. Compared to those with lower-extremity ulceration or amputation, participants with history of peripheral revascularization, were more frequently men and from established market economies (Table 2). They had a shorter duration of diabetes, lower eGFR and serum total cholesterol, were more likely to use lipid lowering and antiplatelet drugs, and to have a history of ever smoking or current alcohol drinking. The cumulative incidence of all-cause mortality, major macrovascular events, cardiovascular death, and fatal and non-fatal myocardial infarction increased comparably in subjects with different presentations of major PAD, compared to those without PAD (Fig. 2; Table 3). Associations of both presentations with all-cause mortality, major macrovascular events, cardiovascular death, and myocardial infarction were confirmed in the fully adjusted model 2. Furthermore, lower-extremity ulceration or amputation was significantly associated with a higher risk of blindness or of requirement for retinal photocoagulation (HR 1.53, 95 % CI 1.01–2.30, p = 0.04). A non-significant trend toward an association of lower-extremity ulceration or amputation was also observed with the risk of major clinical microvascular events (HR 1.44, 95 % CI 0.98–2.11, p = 0.06). However, no association of lower-extremity ulceration or amputation was observed with the risk of ESRD or renal death. The risks for outcomes according to each presentation of major PAD were comparable for mortality and major macrovascular outcomes, but not for retinal complications.

**Table 2:** Clinical characteristics of participants according to different presentations of major PAD at baseline

	Lower-extremity ulceration or amputation (n = 300)	Peripheral revascularization (n = 190)	p
Male sex, n (%)	177 (59.0)	140 (73.7)	0.0009
Region of origin: Asia, n (%)	81 (27.0)	12 (6.3)	<0.0001
Region of origin: established market economies, n (%)	158 (52.7)	135 (71.0)	
Region of origin: Eastern Europe, n (%)	61 (20.3)	43 (22.6)	
Age (years): mean (SD)	66.2 (6.7)	66.6 (6.9)	0.55
Duration of diabetes (years): median (Q1, Q3)	8.0 (4.0, 12.0)	6.0 (3.0, 11.0)	0.007
Waist circumference (cm): mean (SD)	101 (14)	102 (14)	0.72
Body mass index (kg/m^2^): mean (SD)	29.0 (5.9)	28.5 (4.4)	0.32
Heart rate (bpm): mean (SD)	74.5 (11.1)	72.4 (13.2)	0.06
Systolic blood pressure (mmHg): mean (SD)	145 (22)	144 (22)	0.76
Diastolic blood pressure (mmHg): mean (SD)	80 (11)	78 (10)	0.13
Use of antihypertensive treatment, n (%)	210 (70.0)	143 (75.3)	0.21
Disturbance of 10-g monofilament sensation, n (%)	59 (19.7)	30 (15.8)	0.28
Absence of ankle reflex, n (%)	102 (34.0)	51 (26.8)	0.10
Absence of knee reflex, n (%)	54 (18.0)	19 (10.0)	0.02
HbA1c (%): median (Q1, Q3)	7.3 (6.5, 8.7)	7.0 (6.4, 7.9)	0.18
HbA1c (mmol/mol): median (Q1, Q3)	56 (47, 72)	53 (46, 63)	
eGFR (ml/min/1.73 m^2^)	74 (18)	70 (18)	0.01
Urinary albumin to creatinine ratio (µg/mg): median (Q1, Q3)	19 (9, 64)	14 (6, 55)	0.06
Serum total cholesterol (mmol/l): mean (SD)	5.2 (1.1)	4.8 (1.0)	0.0004
Serum HDL cholesterol (mmol/l): mean (SD)	1.2 (0.3)	1.2 (0.3)	0.37
Serum triglycerides (mmol/l): median (Q1, Q3)	1.7 (1.2, 2.5)	1.8 (1.2, 2.3)	0.56
Use of lipid lowering drugs, n (%)	103 (34.3)	126 (66.3)	<0.0001
Use of antiplatelet drugs, n (%)	139 (46.3)	143 (75.3)	<0.0001
History of current smoking, n (%)	47 (15.7)	30 (15.8)	0.97
History of ever smoking, n (%)	153 (51.0)	133 (70.0)	<0.0001
History of current drinking, n (%)	86 (28.7)	89 (46.8)	<0.0001

Comparison of qualitative and quantitative parameters were performed using Chi square and ANOVA tests, respectively. Wilcoxon test was used for variables with skewed distribution (duration of diabetes, HbA1c, urinary albumin-creatinine ratio and triglycerides). p < 0.05 was significant

Asia: Philippines, China, Malaysia, India; Established market economies: Australia, Canada, France, Germany, Ireland, Italy, Netherlands, New Zealand, United Kingdom; Eastern Europe: the Czech Republic, Estonia, Hungary, Lithuania, Poland, Russia, Slovakia. eGFR, estimated Glomerular Filtration Rate computed by the Chronic Kidney Disease Epidemiology Collaboration equation

History of current drinking was defined as consumption of alcohol at least once a week for most weeks of the previous year

**Table 3:** Hazard ratios for outcomes during follow-up according to different presentations of major PAD at baseline

	Absence of PAD	Lower-extremity ulceration or amputation	Peripheral revascularization	Lower-extremity ulceration or amputation (vs. absent PAD)		Peripheral revascularization (vs. absent PAD)	
				HR (95 % CI)	p	HR (95 % CI)	p
All-cause mortality, n (%)	2101 (19.8)	92 (30.7)	63 (33.2)	1.39 (1.12-1.72)	0.003	1.31 (1.01–1.70)	0.04
Major macrovascular events, n (%)	2025 (19.1)	78 (26.0)	56 (29.5)	1.39 (1.11–1.75)	0.005	1.64 (1.25–2.16)	0.0004
Cardiovascular death, n (%)	904 (8.5)	50 (16.7)	29 (15.3)	1.91 (1.43–2.54)	<0.0001	1.52 (1.04–2.23)	0.03
Myocardial infarction, n (%)	665 (6.3)	29 (9.7)	25 (13.2)	1.50 (1.03–2.19)	0.03	1.73 (1.14–2.62)	0.01
Stroke, n (%)	924 (8.7)	24 (8.0)	18 (9.5)	1.02 (0.67–1.55)	0.92	1.54 (0.96–2.48)	0.07
Major clinical microvascular events, n (%)	763 (7.2)	28 (9.3)	11 (5.8)	1.44 (0.98–2.11)	0.06	0.88 (0.48–1.61)	0.68
Retinal photocoagulation or blindness, n (%)	632 (5.9)	24 (8.0)	9 (4.7)	1.53 (1.01–2.30)	0.04	0.94 (0.48–1.83)	0.85
End-stage renal disease or renal death, n (%)	160 (1.5)	4 (1.3)	3 (1.6)	0.84 (0.31–2.30)	0.74	0.94 (0.29–3.02)	0.92

Adjusted as in model 2: region of origin, sex, age, duration of diabetes, body mass index, waist circumference, heart rate, systolic and diastolic blood pressure, disturbance of 10-g monofilament sensation, absence of ankle and knee reflexes, HbA1C, estimated glomerular filtration rate (and its square for macrovascular analyses), total-, and HDL-cholesterol, triglycerides, history of ever smoking and current alcohol drinking, use of antihypertensive, lipid lowering and antiplatelet drugs, and study allocations. p < 0.05 was significant

### Sensitivity analyses

Major PAD was established in 283 (3.7 %) patients among 7679 participants free from history of macrovascular disease at baseline. In this subset of participants, the association of major PAD with the risk of all-cause mortality, major macrovascular events and cardiovascular death remained significant (Additional file 1: Table S2). Lower-extremity ulceration or amputation, and the history of peripheral revascularization also remained significantly associated with increased risk for all-cause mortality (HR 1.60, 95 % CI 1.22–2.10, p = 0.0007, and HR 1.57, 95 % CI 1.06–2.34, p = 0.02, respectively) and major macrovascular events (HR 1.63, 95 % CI 1.20–2.21, p = 0.002, and HR 1.80, 95 % CI 1.14–2.86, p = 0.01, respectively).

## Discussion

We have investigated the risk of vascular outcomes with major PAD, and its different presentations, in patients with type 2 diabetes followed for 10 years in the ADVANCE and ADVANCE-ON studies. Major PAD at baseline was associated with increased risk of all-cause mortality, major macrovascular events, cardiovascular death, and myocardial infarction. Lower-extremity ulceration or amputation, and the history of peripheral revascularization displayed similar risks of death and major cardiovascular events. However, lower-extremity ulceration or amputation, but not peripheral revascularization, was also associated with increased risk of retinal photocoagulation or blindness.

### History of PAD and risk of macrovascular events

In line with our findings, poor survival and macrovascular outcomes associated with PAD were reported in other prospective studies of patients with type 2 diabetes, even in those who have not been treated with hypoglycaemic drugs [13–15, 20]. In the PROspective pioglitAzone clinical trial in macrovascular events (PROactive) trial, baseline PAD was associated with high risk of all-cause mortality, and macrovascular complications in 5238 patients with type 2 diabetes during a mean follow-up of 2.9 years [14]. In the Bypass Angioplasty Revascularization Investigation 2 Diabetes (BARI 2D) trial, low ankle-brachial index or non-compressible artery were associated with increased risks of death and major cardiovascular events in 2368 patients with type 2 diabetes followed for an average of 4.3 years [15]. Our larger study demonstrates that the association of major PAD with death and major macrovascular events persisted during 10 years of follow-up, even after adjustment for major risk factors. Furthermore, these associations persisted in patients free for any history of macrovascular disease at baseline.

### Presentations of PAD and risk of macrovascular events

As distal PAD is known to be associated with a high rate of ulceration and amputation, and is rarely accessible for revascularization [9, 21], it is tempting to speculate that our patients with history of lower-extremity ulceration or amputation, were more likely to have a distal than a proximal origin for their PAD. The determination of PAD localizations differs among studies, but angiography and Doppler ultrasound are still considered as the key imaging methods [21, 22]. A recent study has reported a semi-quantitative ultrasonographic score to evaluate the association of PAD severity and localizations with cardiovascular risk factors and adverse events [23]. This showed that the magnitude of the association of PAD with diabetes was greater with distal than proximal localizations. Major PAD presentations had different clinical characteristics in our study. Lower-extremity ulceration or amputation was more common than peripheral revascularization, and associated with a longer duration of diabetes. Furthermore, lower-extremity ulceration, amputation, and peripheral revascularization occurred concurrently in only 26 patients, suggesting that these presentations of major PAD have different origins. Comparison of survival, macrovascular and microvascular outcomes according to different presentations of major PAD have not been extensively performed in people with diabetes. Despite decrease in its prevalence and incidence during last decades in some regions in the world [24, 25], lower-extremity amputation will still exert an important health burden. Lower-extremity amputation and ulceration were associated with increased risk of major macrovascular events, and of cardiovascular and non-cardiovascular death in patients with diabetes [26–29]. The risk of major cardiovascular events was also high in patients with PAD undergoing peripheral revascularization in a general population cohort including 39 % of participants with diabetes [30]. The pathophysiological links by which PAD might predispose to poor vascular outcomes have not yet been fully elucidated in patients with diabetes. The increased formation of advanced glycation end-products (AGE) and its receptors (RAGE) may be a potential pathway linking PAD and other major vascular outcomes. A recent study has shown association of high plasma levels of AGE-RAGE components with increased risk for amputation, PAD, or death in patients with type 2 diabetes [31].

### Presentations of PAD and risk of microvascular events

Importantly, our findings highlight for the first time, the association of lower-extremity ulceration or amputation with the risk of severe eye complications during 10-years follow-up. This is comparable to a recent cross-sectional study, which showed a higher prevalence of PAD in patients with proliferative diabetic retinopathy rather than those with non-proliferative retinopathy [32]. We have also reported recently, association of history of diabetic retinopathy with requirement for laser photocoagulation at baseline with excess risk of major PAD during follow-up in ADVANCE study [33]. Our present findings are unlikely to be explained by a possible effect of peripheral diabetic neuropathy, since associations we have observed persisted despite adjustment for disturbance of 10-g monofilament sensation and absence of ankle and knee reflexes. Furthermore, the distribution of the key neuropathic markers was comparable between the different presentations of major PAD. However, we did not observe an association of either presentation of major PAD with the risk of major renal outcomes. Remarkably, only 3 patients with major PAD at baseline developed ESRD during 10 years of follow-up. Based on their high rate of mortality, patients with both PAD and chronic kidney disease (CKD) may have died before experiencing more advanced renal outcomes [34, 35]. A recent study showed that the 4-year mortality rate of patients with PAD and CKD stages 4, and 5 was 72 and 78 %, respectively [36].

### Strengths and limitations

As compared to previous studies in people with type 2 diabetes, we have observed a lower prevalence of PAD, which may partially be explained by the differences in PAD definitions used [13, 14]. The main limitation of our investigation is that we have studied patients with symptomatic and severe PAD, and may have underestimated outcomes associated with asymptomatic PAD. However, our work has several strengths including the investigation of a large contemporary cohort of patients with type 2 diabetes at high cardiovascular risk, with a comprehensive clinical history of major PAD at baseline, and pre-specified major endpoints during the study period.

## Conclusions

Our study underlines worse survival and cardiovascular outcomes in patients with type 2 diabetes and major PAD during 10 years of follow-up. Lower-extremity ulceration or amputation, and peripheral revascularization had similar effects on the risk of these outcomes. Furthermore, lower-extremity ulceration or amputation was associated with higher incidence of severe eye disease. Prompt screening and more intensive management of both presentations of major PAD may help to improve prognosis, particularly cardiovascular outcomes in patients with long-standing type 2 diabetes.

## Additional file

10.1186/s12933-016-0446-x Clinical characteristics of participants by history of major PAD at baseline. **Table S2.** Relative risk for outcomes during follow-up according to major PAD at baseline in participants free from history of macrovascular disease at baseline.

## Abbreviations

ADVANCE: action in diabetes and vascular disease—preterax and diamicron modified-release controlled evaluation trial
AGE: advanced glycation end-products
ANOVA: analysis of variance
BARI 2D: bypass angioplasty revascularization investigation 2 diabetes trial
CI: confidence interval
CKD: chronic kidney disease
CKD-EPI: chronic kidney disease–epidemiology collaboration equation
eGFR: estimated glomerular filtration rate
ESRD: end stage renal disease
HR: hazard ratio
PAD: peripheral arterial disease
PROactive: PROspective pioglitAzone clinical trial in macrovascular events trial
RAGE: receptor of advanced glycation end-products
SD: standard deviation
