# Supplementary material for: Presentations of major peripheral arterial disease and risk of major outcomes in patients with type 2 diabetes: results from the ADVANCE-ON study
Source: Cardiovasc Diabetol. 2016 Sep 2;15(1):129. doi: 10.1186/s12933-016-0446-x (PMC5010714; doi:10.1186/s12933-016-0446-x)
Supplement: Supplementary file 1 — 10.1186/s12933-016-0446-x Clinical characteristics of participants by history of major PAD at baseline. Table S2. Relative risk for outcomes during follow-up according to major PAD at baseline in participants free from history of macrovascular disease at baseline. [file 12933_2016_446_MOESM1_ESM.docx]

**Additional Table S1. Clinical characteristics of participants by history of major PAD at baseline**

|  | Overall  (n=11140) | History of major PAD at baseline | | P |
| --- | --- | --- | --- | --- |
|  |  | No  (n=10624) | Yes  (n=516) |  |
| Male sex, n (%) | 6407 (57.5) | 6068 (57.1) | 339 (65.7) | 0.0001 |
| Region of origin: Asia, n (%) | 4136 (37.1) | 4040 (38.0) | 96 (18.6) | <0.0001 |
| Region of origin: Established market economies, n (%) | 4862 (43.7) | 4547 (42.8) | 315 (61.0) |  |
| Region of origin: Eastern Europe, n (%) | 2142 (19.2) | 2037 (19.2) | 105 (20.4) |  |
| Age (years): mean (SD) | 65.7 (6.4) | 65.7 (6.4) | 66.4 (6.7) | 0.02 |
| Duration of diabetes (years): median (Q1, Q3) | 7.0 (3.0, 11.0) | 7.0 (3.0, 11.0) | 7.0 (4.0, 12.0) | 0.20 |
| Waist circumference (cm): mean (SD) | 99 (13) | 98 (13) | 102 (14) | <0.0001 |
| Body mass index (kg/m^2^): mean (SD) | 28.3 (5.2) | 28.3 (5.2) | 28.8 (5.4) | 0.02 |
| Heart rate (bpm): mean (SD) | 74.1 (12.1) | 74.1 (12.1) | 73.8 (12.0) | 0.49 |
| Systolic blood pressure (mmHg): mean (SD) | 145 (21) | 145 (21) | 144 (22) | 0.55 |
| Diastolic blood pressure (mmHg): mean (SD) | 81 (11) | 81 (11) | 79 (11) | 0.0003 |
| Use of antihypertensive treatment, n (%) | 7655 (68.7) | 7281 (68.5) | 374 (72.5) | 0.06 |
| Disturbance of 10-g monofilament sensation, n (%) | 988 (8.9) | 895 (8.4) | 93 (18.0) | <0.0001 |
| Absence of ankle reflex, n (%) | 2401 (21.6) | 2234 (21.0) | 167 (32.4) | <0.0001 |
| Absence of knee reflex, n (%) | 1011 (9.1) | 933 (8.8) | 78 (15.1) | <0.0001 |
| HbA1c (%): median (Q1, Q3) | 7.2 (6.5, 8.2) | 7.2 (6.5, 8.2) | 7.2 (6.4, 8.3) | 0.58 |
| HbA1c (mmol/mol): median (Q1, Q3) | 55 (47, 66) | 55 (47, 66) | 55(46, 67) |  |
| eGFR (ml/min/1.73 m^2^) | 74 (17) | 74 (17) | 72 (18) | 0.002 |
| Urinary albumin-creatinine ratio (µg/mg): median (Q1, Q3) | 15 (7, 40) | 15 (7, 39) | 18 (7, 62) | 0.02 |
| Serum total cholesterol (mmol/l): mean (SD) | 5.2 (1.2) | 5.2 (1.2) | 5.0 (1.1) | 0.001 |
| Serum HDL cholesterol (mmol/l): mean (SD) | 1.3 (0.4) | 1.3 (0.4) | 1.2 (0.3) | 0.03 |
| Serum triglycerides (mmol/l): median (Q1, Q3) | 1.6 (1.2, 2.3) | 1.6 (1.2, 2.3) | 1.7 (1.2, 2.4) | 0.15 |
| Use of lipid lowering drugs, n (%) | 3934 (35.3) | 3689 (34.7) | 245 (47.5) | <0.0001 |
| Use of antiplatelet drugs, n (%) | 5199 (46.7) | 4896 (46.1) | 303 (58.7) | <0.0001 |
| History of current smoking, n (%) | 1550 (13.9) | 1469 (13.8) | 81 (15.7) | 0.23 |
| History of ever smoking, n (%) | 4674 (42.0) | 4369 (41.1) | 305 (59.1) | <0.0001 |
| History of current drinking, n (%) | 3396 (30.5) | 3211 (30.2) | 185 (35.9) | 0.007 |

Comparison of qualitative and quantitative parameters were performed using Chi-square and ANOVA tests, respectively. Wilcoxon test was used for variables with skewed distribution (duration of diabetes, HbA1c, urinary albumin-creatinine ratio and triglycerides). p<0.05 was significant.

Asia: Philippines, China, Malaysia, India; Established market economies: Australia, Canada, France, Germany, Ireland, Italy, Netherlands, New Zealand, United Kingdom; Eastern Europe: the Czech Republic, Estonia, Hungary, Lithuania, Poland, Russia, Slovakia. eGFR, estimated Glomerular Filtration Rate computed by the Chronic Kidney Disease Epidemiology Collaboration equation.

History of current drinking was defined as consumption of alcohol at least once a week for most weeks of the previous year.

**Additional Table S2. Relative risk for outcomes during follow-up according to major PAD at baseline in participants free from history of macrovascular disease at baseline**

|  | History of major PAD at baseline | | Hazard ratios (major PAD *vs.* not) | |
| --- | --- | --- | --- | --- |
|  | No  (n=7396) | Yes  (n=283) | HR  (95% CI) | P |
| All-cause mortality, n (%) | 1294 (17.5) | 88 (31.1) | 1.52 (1.22 – 1.91) | 0.0003 |
| Major macrovascular events, n (%) | 1111 (15.0) | 66 (23.3) | 1.59 (1.23 – 2.06) | 0.0004 |
| Cardiovascular death, n (%) | 473 (6.4) | 39 (13.8) | 2.04 (1.46 – 2.85) | <0.0001 |
| Myocardial infarction, n (%) | 353 (4.8) | 22 (7.8) | 1.41 (0.90 – 2.21) | 0.13 |
| Stroke, n (%) | 502 (6.8) | 24 (8.5) | 1.47 (0.94 – 2.30) | 0.09 |
| Major clinical microvascular events, n (%) | 503 (6.8) | 23 (8.1) | 1.19 (0.78 – 1.81) | 0.43 |
| Retinal photocoagulation or blindness, n (%) | 420 (5.7) | 17 (6.0) | 1.11 (0.68 – 1.82) | 0.67 |
| End-stage renal disease or renal death, n (%) | 104 (1.4) | 7 (2.5) | 1.30 (0.58 – 2.89) | 0.52 |

Adjusted as in model 2: region of origin, sex, age, duration of diabetes, body mass index, waist circumference, heart rate, systolic and diastolic blood pressure, disturbance of 10-g monofilament sensation, absence of ankle and knee reflexes, HbA1C, estimated glomerular filtration rate (and its square for macrovascular analyses), total-, and HDL-cholesterol, triglycerides, history of ever smoking and current alcohol drinking, use of antihypertensive, lipid lowering and antiplatelet drugs, and study allocations. p<0.05 was significant.
